# Association of triclosan and human infertility: A systematic review

**DOI:** 10.5620/eaht.2023015

**Published:** 2023-06-30

**Authors:** Belén Daza-Rodríguez, Dilia Aparicio-Marenco, Johana Márquez-Lázaro

**Affiliations:** 1 Medicine program, Corporación Universitaria Rafael Núñez, Cartagena, Colombia; 2 GINUMED group, Medicine program, Corporación Universitaria Rafael Núñez, Cartagena, Colombia; 3 TOXSA group, Medicine program, Corporación Universitaria Rafael Núñez, Cartagena, Colombia

**Keywords:** Triclosan, infertility, men, women, semen quality, ovarian reserve

## Abstract

Triclosan (TCS) is a chemical compound, which has antibacterial, antiviral, and antifungal properties. TCS is considered an endocrine-disrupting chemical, which has been shown to interfere with developmental, behavioral, and reproductive outcomes in biological models and cell cultures. However, implications about exposure to TCS and human infertility are rare. Thus, the main of this review is summarize the available evidence of the association between triclosan exposure on human infertility. For this, systematic review was conducted following the recommendations established in Report of Systematic Reviews and Meta-Analyses guide (PRISMA). Initially, an electronic search in MEDLINE (via PubMed) and Science direct was performed. The methodological quality of the included studies was verified through the Joanna Briggs Institute (JBI) checklists. All selection and data extraction processes were carried out independently by two reviewers. The evidence was organized and presented using tables and narrative synthesis. There is lacking evidence about the association between triclosan and human infertility. Overall, no association between triclosan and infertility was found. However, semen quality and ovarian reserve are susceptible to triclosan exposure. Thus, future studies are still needed to better elucidate the associations between triclosan and infertility outcomes.

## Introduction

Personal care products such as soaps, toothpaste, mouthwashes and deodorants or cosmetics are widely used around the world for personal hygiene and beautification. These products contain some substances that help to preserve them, such as triclosan [1]. Triclosan (TCS) is a chemical compound, which has antibacterial, antiviral, and antifungal properties [2]. Also, TCS is employed in kitchen utensils, electronics, plastics, toys, clothes, and bedclothes, as well as disinfectants, surgical sutures antiseptics, implants, and medical devices [2, 3].

Currently, TCS is considered a high potential endocrine-disrupting chemical because this has been shown to interfere with developmental, behavioral, and reproductive outcomes in biological models [4–6]. Animal studies have shown that triclosan can disrupt hormone regulation and reproductive function. For example, some studies on male rats and male mice have found that triclosan exposure can lead to changes in hormone levels, reduced sperm production, and impaired reproductive organ development [7–12]. Also, studies in female zebrafish showed that TCS exposure promoted ovarian oxidative damage and accelerated ovarian cell apoptosis while mice and rats cause downregulated thyroid hormone levels, hyperprolactinemia, and restrain hypothalamic kisspeptin expression [13–15]. These events could promote reproductive endocrine disturbance in these animals [16]. However, the evidence regarding a direct link between triclosan exposure and fertility issues is inconclusive.

In terms of human studies, research is ongoing, and the current evidence is limited. While the direct link between triclosan exposure and infertility has not been definitively established, there have been some studies that suggest potential associations between triclosan exposure and various reproductive health outcomes in humans [17]. In this sense, studies have indicated a potential association between triclosan exposure and altered hormone levels, pubertal development, increased body mass index (obesity), reduced fecundability (the ability to conceive), decreased ovarian reserve, decreased sperm concentration, increased risk of spontaneous abortion, and decreased sperm count and morphology [6, 18]. Hence, Food and Drug Administration (FDA) banned the use of triclosan in over-the-counter antiseptic wash products due to concerns over its safety and effectiveness, however, it is still employed in toothpaste, mouthwash, hand sanitizer, and surgical soap manufacturing [3, 19].

Triclosan is indeed a widespread environmental contaminant, and its presence has been detected in various environmental and biological samples [3]. Studies have shown that TCS can be found in urine, blood, breast milk, amniotic fluid, and other biological matrices from individuals in different parts of the world [20]. Human exposure to TCS can occur through dermal contact, such as when using personal care products containing triclosan, as well as through direct ingestion when consuming food or water that has been contaminated with triclosan. The use of triclosan in various consumer products and its subsequent release into the environment contribute to its presence in water bodies and the food chain [20, 21].

Besides, the infertility is a significant concern for many individuals and couples around the world, and researchers have sought to explore the potential links between environmental factors and reproductive outcomes. Thus, the objective of this is systematic review was to summarize the available evidence of the association between triclosan exposure and human infertility.

## Methods

### Type of study

A systematic review according to the guidelines of the PRISMA statement (Preferred Reporting Items for Systematic Reviews and Meta-Analyzes) was carried out [22]. The PRISMA statement consists of a checklist that authors can use to ensure they have included all the necessary information in their systematic review or meta-analysis. By following the PRISMA guidelines, researchers can enhance the transparency and reliability of their work, making it easier for readers to assess and interpret the findings. The review protocol has been registered prospectively in PROSPERO (Registration number PROSPERO 2022: CRD42022340296).

### Search strategy

A systematic electronic search of the literature was done in MEDLINE (PubMed) and Science direct database. The search objective was to identify studies that evaluated the effects of triclosan on human infertility from 2011 to 2021. Search strategies were adapted to each database, using the following keywords: (triclosan) OR (TCS) OR (5-chloro-2-(2,4-dichlorophenoxy) phenol) AND ("Fertility"[Mesh]) OR ("Infertility"[Mesh]) OR (menstrual cycle) OR (reproductive health) OR (semen) OR (gametogenesis) OR (sperm*) OR (Puberty) OR (spermatogenesis) OR (testis) OR (ovary) OR (endometriosis) OR (ovarian reserve) OR (polycystic ovary syndrome) OR (oocyte). The update of the search was performed on February 4, 2022.

### Study selection

Only studies meeting the inclusion criteria were selected: (a) research evaluating the effect of triclosan on infertility; (b) studies conducted in a human population; (c) cohort, case-control, and cross-sectional studies, (d) studies published between 2011 to 2021. All experimental reports, book chapters, reviews, in silico studies, conference abstracts, and editor letters were excluded.

All the reports obtained were stored in the Rayyan platform [23] and once the duplicates had been eliminated, titles and abstracts were independently examined by two reviewers, considering the inclusion and exclusion criteria. Then, the full text of all the papers was read, and only studies meeting the inclusion criteria were selected. When there was any disagreement between the two reviewers, a third reviewer was included in the decision-making process.

### Methodological quality assessment

The studies included in this review were assessed using the Joanna Briggs Institute (JBI) checklist [24]. In general, these checklists evaluate the quality of different aspects such as the selection process, measurements, and comparability of the groups. This instrument has 11 and 10 points of the total score for cohort studies and case-control studies, respectively. These checklists do have not cut-off points, but a higher score is indicative of better methodological quality. The process was carried out by two reviewers independently and when there was any disagreement between the two reviewers, a third reviewer was included in the decision-making process.

### Data extraction and analysis

The following data were extracted from each article: general characteristics of the studies (authors, country, sample size, sex of sample (women or men), and aspects related to exposure to triclosan and its effect on infertility (type of sample, triclosan concentration, assay type, odds ratio (OR)) and main results. Given the degree of heterogeneity between the studies, the data collected in this systematic review could not be used for meta-analysis purposes. For this reason, a description of the findings and a narrative synthesis of the evidence were carried out.

## Results and Discussion

### Selection of studies

**Figure 1.**
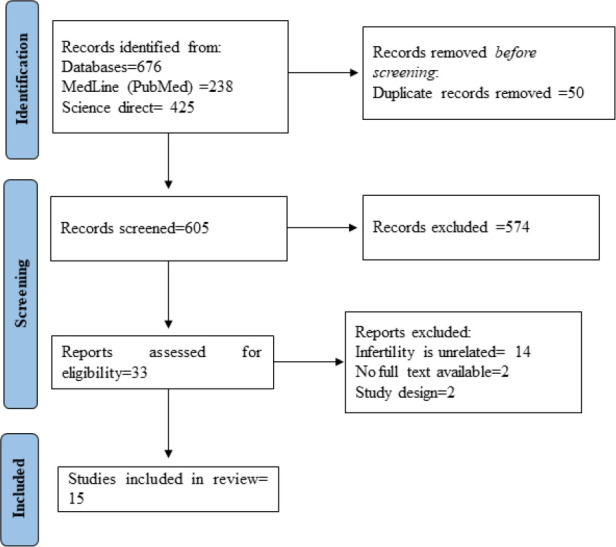
PRISMA flowchart describing the selection of studies.

A total of 676 studies were identified following the search strategy; however, 50 were duplicates. Once removed, titles and abstracts were screened and 33 were selected for full-text selection. Of these, 16 did not meet the inclusion criteria, and 15 [25–39] studies were finally selected for the review, Figure 1.

### Characteristics of the selected studies

The 15 [25–39] studies selected for this review were published in English between 2011 and 2021. These were six studies from United States [25, 26, 33–36], six from China [27, 29, 30, 37–39], two from Poland [31, 32] and one from Belgium [28]. There were nine cohort studies [26, 30–36, 38], three were case and control studies [27–29], and three were cross-sectional studies [25, 37, 39]. Eight studies were carried out in men [26–28, 31, 34–37] and seven in women [25, 29, 30, 32, 33, 38, 39]. The age varied from 18 [25, 26] to 36 years [33, 34], which is known as reproductive age. The men sample varied from 40 to 877 [27, 28] while the women sample between 40 to 895 [25, 29]. In all studies, urine was used for the quantification of triclosan. The use of urine as sample is based in that TCS is primarily excreted through in this biological fluid, regardless of the route of exposure, and has a relatively short elimination half-life of approximately 11 hours. Also, due to its efficient excretion via urine, measuring TCS levels in urine provides a reliable and timely indication of recent TCS exposure form personal care products and environmental sources [16, 17]. The characteristics of the selected studies are presented in Table 1.

### Methodological quality of the included studies

**Figure 2.**
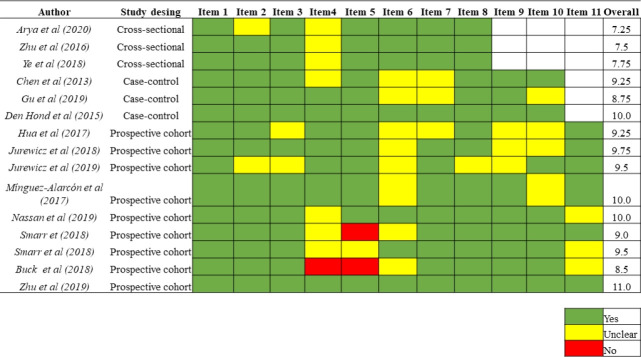
The methodological quality of the included studies by design.

Using the JBI´s critical appraisal checklist tool, the mean score was 9.6 ± 0.71 (8.5 to 11), 9.3 ± 0.63 (8.8 to 10), and 7.5± 0.25 (7.3 to 7.8) for cohort, case-control, and cross-sectional studies, respectively. The results in detail for each study are shown in Figure 2.

### Triclosan and men´s infertility

Overall, included studies show a variety of assays to evaluate the triclosan effect on human reproductivity. Eight studies evaluated the effect of TCS on men's infertility [26–28, 31, 34–37]. TCS concentration was measured in urine [27, 28, 31, 34, 35, 37] or urine and semen samples [26, 36]. Also, semen samples were used to evaluate quality parameters [27, 28, 31, 34–37] while blood was used to measure reproductive enzyme activities [28] The urine concentrations of TCS varied from 0.5x10-3 [31] to 17.75 ng/mL [35], while semen samples between 10.61[26] to 12.6 ng/mL [36]. The effect of triclosan on men's fertility is summarized in Table 2.

In selected studies, only three evaluated the direct association between triclosan and infertility [26–28], while others through semen quality parameters [31, 34–37]. In this sense, Buck et al., Chen et al., and Den Hond et al., found no association between TCS exposure and infertility [26–28], however, Den Hond et al., indicate that triclosan could induce alterations in fertility in adulthood, through hormonal disruption [28].

Regarding the effects of triclosan exposure on semen quality, Zhu et al., provides evidence that exposure to TCS is associated with poorer semen quality (sperm volume, total sperm count, sperm motility, sperm morphology) [37]. This finding was supported by Jurewicz et al., which found a sperm with abnormal morphology [31]. However, Smarr et al., in two studies did not find a relationship between triclosan exposure and a decrease in semen quality [35, 36]. Also, they found that triclosan exposure was suggestive of enhanced sperm morphometry and morphology [35]. Nassan et el., either did not observe any significant dose-response trend between urinary triclosan concentrations and semen quality parameters [34]. However, they found that detectable concentrations of TCS have a consistent pattern of lower percent morphologically normal sperm compared to undetectable concentrations [34].

Overall, the measurement of semen |quality parameters can be considered an important marker for the evaluation of the effects of endocrine-disrupting chemicals on reproductive outcomes including men´s infertility.

### Triclosan and women's infertility

The effect of triclosan on women's reproductivity was evaluated in different ways. In this sense, seven studies were selected [25, 29, 30, 32, 33, 38, 39]. In all studies, the TCS concentration was measured in urine [25, 29, 30, 32, 33, 38, 39]. The urine concentrations of TCS varied from 0.25 [39]to 13.0 ng/mL [33]. The effect of TCS on women´s reproductive outcomes was studied through its association with infertility [25, 38], subfertility [30], polycystic ovary syndrome (PCOS) [29, 39] and antral follicle count (AFC) [32, 33]. These effects are summarized in Table 3.

Zhu et al., did not find an association between TCS concentration and risk of infertility, however, they observed a relationship between risks of abnormal menstruation and reduced fecundity [38]. In this sense, Arya et al., did not find either association between TCS concentration and infertility, but higher parity or gravidity levels were associated with lower TCS levels (p=0.001) [25]. Otherwise, Hua et al., did not observe a dose-response correlation between TCS concentrations and the outcomes of fertilization, however, TCS was associated with negative effects on embryo formation (OR = 1.332, p:0.020) and implantation (OR = 1.709, p:0.040) [30]. Also, in a recent study not included in this review, reported that TCS exposure could be a plausible risk factor for fertility in women of reproductive age [40].

With regard, to polycystic ovary syndrome (PCOS) and triclosan exposure, Ye et al., found a positive association in infertile women. However, Gu et al., did not find a significant association between these variables [29].

On the other hand, Míngez et al., found that triclosan concentrations were inversely associated with AFC. Also, this association was stronger among younger and lean women [33]. This observation was also reported by Jurewicz et al., which was held when the model adjusted for age, smoking, and body mass index [32].

In general, infertility could be derived from problems in the menstrual cycle, fecundity, fertilization, PCOS, and ovarian reserve. Thus, these factors can be considered an indirect form for the measurement of women's reproductive outcomes derived from exposure to environmental pollutants, which are implicated in the fertility of women.

Currently, the fertility rate around the world is decreasing. This problem can be attributed to different factors, including environmental pollutants, which have been increasingly detected in humans [41,42]. Due to its widespread cumulative burden, the effects on reproductive health even at low-level exposures are a growing concern, especially with Endocrine-Disrupting Chemicals (EDCs) [43]. This group belongs to triclosan, which has been associated with decreases in fertility in animals and humans [44], therefore, to describe the effects of triclosan exposure on human infertility, we conducted a literature-based comprehensive analysis.

Overall, our findings suggest that non-occupational exposure to triclosan may not have an effect directly on women's and men's infertility [25–28, 38]. However, TCS exposure could affect men´s fertility by a decrease in semen quality parameters [26, 31, 34, 37], while women´s fertility by antral follicle count [32, 33].

According to selected studies in men, exposure to triclosan can affect semen quality through a decrease in sperm volume, sperm count, and sperm motility as well as changes in sperm morphology [26, 31, 34, 37]. These observations were like those reported by Zamkowska et al., however, they only included three studies due to little published evidence at the time [45]. Nevertheless, considering the insufficient evidence, further epidemiological studies are needed to confirm these findings.

Regarding selected studies in women, triclosan exposure could influence the decrease of antral follicle count, which is a well-accepted marker of ovarian reserve; one predictor of women's fecundity, however, evidence is not enough [32, 33]. In this sense, our findings are like those reported by Minguez et al., who have also indicated that the lack of evidence difficult to make conclusions regarding the effect of women's exposure to TCS on fecundity [18]. Also, Maksymowicz et al., indicated in a review that includes animal and human studies that TCS exposure could lead to subfertility, but more studies are necessary [46].

Some limitations of the reviewed studies included the heterogeneity regarding population, intervention, design, and results, which can indeed make it challenging to effectively combine and synthesize data. This heterogeneity can stem from variations in study populations (such as age, geographic location, and underlying health conditions), differences in exposure levels and duration, diverse study designs (observational, experimental, cohort studies), and varying outcomes measured. Furthermore, since infertility is a complex condition influenced by multiple factors such as genetics, lifestyle, environmental exposures, and overall health, establishing causality can be challenging. In the order hand, these studies have limited data on long-term effects; thus, it is not possible to know the real scenario of effects of triclosan on human reproduction. In addition, the presence of confounding factors as hormonal imbalances, underlying medical conditions, lifestyle choices, and exposure to other environmental chemicals could making it difficult to attribute infertility solely to triclosan exposure.

The main practical implications of this review are related to showing research and physicians the deleterious effects of triclosan exposure in human infertility according to epidemiological studies published; considering that this disease has become a serious social, mental, and physical health problem worldwide. Therefore, physicians should consider and/or incorporate exposure to environmental pollutants such as TCS another possible trigger for fertility problems in both women and men. In addition, the reviewed studies examined the potential association between triclosan and reproductive effects in humans, which contributes significantly to the field of toxicology by providing valuable information on the reproductive toxicity of this chemical compound. Also, these studies highlight the importance of considering the potential adverse effects of widely used chemicals on human health and provide a basis for further research and regulatory actions to protect public well-being.

Some limitation of this review could be the language barrier because all studies were published in English, which eliminated the evidence published in any other language. Nevertheless, no restrictions about languages were carried out; moreover, since most evidence is published in English, it is easier for evidence in this language to meet the criteria established for the search. Among the strengths of this review, we highlight that all methods were described in a PROSPERO protocol, and the selection, and methodological quality assessment process was carried out by at least two reviewers independently, which provides reasonable confidence in our results.

## Conclusions

Although the epidemiological literature on the effects of EDCs on the reproductive outcome is growing, the evidence supporting an association between triclosan, and human infertility remains unclear. The heterogeneity of results in the selected studies could be due to methodological differences (sample size, sample type), study designs, measurement of TCS concentration (analytical methods), endpoints, and confounding factors (diet, age, body mass index). Therefore, future studies are still needed to further elucidate the associations between environmental exposure to triclosan and human fertility.

## Figures and Tables

**Table 1.** Characteristics of studies included in the review.

Reference	Type of study	Country	Population	Age (years)	Sample
[25]	Cross-sectional	United States	Women	18-45	895
[26]	Prospective cohort	United States	Men	>18	339
[27]	Case-control	China	Men	Control: 29.83 ± 3.86;	Control: 713
				Cases: 28.50 ± 4.35	Cases: 877
[28]	Case-control	Belgium	Men	Control: 31.6 (29.0; 35.0);	Control: 80
				Cases: 34.1 (30.0; 38.5)	Cases: 40
[29]	Case-control	China	Women	Control: 29.8 ± 3.2;	Control: 83
				Cases: 30.5 ± 3.6	Cases: 40
[30]	Prospective cohort	China	Women	31.4 ± 4.4 (21–39)	144
[31]	Prospective cohort	Poland	Men	32.14 ± 4.2	315
[32]	Prospective cohort	Poland	Women	33.30 ± 3.69	511
[33]	Prospective cohort	United States	Women	Median: 36	109
[34]	Prospective cohort	United States	Men	36.6 ± 5.24	262
[35]	Prospective cohort	United States	Men	31.8 ± 4.9	501
[36]	Prospective cohort	United States	Men	31.8 ± 4.9	439
[37]	Cross-sectional	China	Men	30.8 ± 4.1	471
[38]	Prospective cohort	China	Women	29.8 ± 3.0	698
[39]	Cross-sectional	China	Women	PCOS: 30.4 ± 3.6;	PCOS: 84;
				No-PCOS: 32.2 ± 4.2	No-PCOS: 212

PCOS: Polycystic ovary syndrome.

**Table 2.** Summary of evidence on TCS exposure and effect on men's infertility.

Reference	Biological sample	Age (years)	TCS concentration	Assays	OR	Outcome
[26]	Urine and semen	>18	Seminal plasma: 0.043 ng/mL	TTP	FORs: 1.09 (0.94 - 1.26)	Although FORr >1, no significance was found between the presence of TCS and the fertility of the couple measured as TTP.
			Semen: 10.61 ng/mL;			
			Urine: 17.75 (Median) ng/mL			
[27]	Urine and semen	Control: 29.83 ± 3.86	Control: 1.593 ng/mL	SV, SC, and SN	1.27 (0.59 – 2.70)	No significant association was found between TCS and idiopathic infertility, and abnormal semen parameters
		Cases: 28.50 ± 4.35	Cases: 1.707 ng/mL			
			-Geometric mean			
[28]	Urine semen blood	Control: 31.6 (29.0; 35.0)	Control: 2.8 (0.5; 5.5) μg/L;	SV, SC, and SM Total testosterone, LH, FSH, SHBG, total 17b-oestradiol,andinhibin B.	0.99 (0.62; 1.59)	No relationship was found between TCS and subfertility. However, TCS levels were positively related to LH, and negatively toInbin B concentrations.
		Cases: 34.1 (30.0; 38.5)	Cases: 2.6 (0.5; 15.6) μg/L			
[31]	Urine and semen	32.14 ± 4.2	0.506 – 789.20 μg/L	SCS, SM, morphology, CASA sperm, DNA damage, and total sperm disomy		Positive associations were observed between the urinary concentrations of TCS 50th–75th percentile and the percentage of sperm with abnormal morphology (*p = 0.016*) compared to urinary concentrations of TCS <25th percentile.
[34]	Urine and semen	36.6 ± 5.24	0.68 (0.61 – 0.74) ng/mL	Sperm morphology, SN and SM		No significant association was observed between triclosan and semen quality parameters.
			-Geometric mean			
[35]	Urine and semen	31.8 ± 4.9	17.6 (4.42, 77.1) ng/mL	Sperm viability, SM, sperm morphometry, and sperm DNA damage.		No association was found between TCS concentrations and semen quality parameters.
[36]	Semen	31.8 ± 4.9	12.6 (10.4, 15.1) ng/mL	Sperm viability, SM, sperm morphometry, and sperm DNA damage.	Volume: 0.83 (0.57, 1.22), p=0.67.	No association was found between TCS concentrations and semen quality parameters.
					Sperm Concentration: 0.97 (0.69, 1.38), p=0.98.	
					Total Count: 0.98 - 1.24 (0.82, 1.87), p=0.67.	
					Sperm Viability: 0.91 (0.71, 1.16), p=0.73.	
					DNA Fragmentation: 1.13 (0.76, 1.66), p=0.78.	
[37]	Urine and semen	30.8 ± 4.1	0.99 ng/mL	SV, SC, SN, SM, sperm speed, sperm morphology and Average path Velocity.		TCS was negatively associated with sperm concentration, sperm count, number of forward moving sperms, and percentage and number of normally morphologic sperms.

TCS: Triclosan; FORs: fecundability odds ratios; TTP: time to pregnancy; SV: semen volume; SC: sperm concentration; SN: sperm number; SM: sperm motility; CASA: VSL straight-line velocity; VCL: curvilinear velocity, LIN linearity; FSH: follicle-stimulating hormone; LH: luteinizing hormone.

**Table 3.** Summary of evidence on TCS exposure and effect on women's infertility.

Reference	Biological sample	Age (years)	TCS concentration	Assays	OR	Outcome
[25]	Urine	18-45	Infertile: 66.83 ± 15.30 ng/mL	The concentration of triclosan in the urine	PR mixture: 1.13 (CI: 1.04 - 1.24), *p=0.007*	No association was found between TCS exposure and self-reported infertility.
			Fertile: 57.13 ± 8.19 ng/mL			
[29]	Urine	Control: 29.8 ± 3.2	Control and cases < LOD (0.15 – 53.6 μg/L).	The concentration of triclosan in urine.	PCOS, OR: 0.869 (0.625, 1.183), *p:0.354*	No significant association was found betweenTCS and PCOS.
		Cases: 30.5 ± 3.6				
[30]	Urine and blood	31.4 ± 4.4 (21–39)	0.358 ± 0.728 urinary (μmol/mol Creatinine)	Concentration of triclosan in urine	OR: 1,238 (0.990 – 1.548), *p:0.061*	No association was observed between TCS concentrations and fertilization outcomes on women with subfertility.
[32]	Urine and blood	33.30 ± 3.69	3.04 ± 7.14 μg/L (creatinine)	AFC, AMH, FSH and E2		Urinary triclosan concentrations were negatively associated with antral follicle count (*p = 0.03*) in model. However, no statistical difference between ovarian reserve parameters (AMH, FSH, estradiol level), and triclosan exposure were found.
[33]	Urine	Median:36	13.0 (Geometric mean) μg/L (creatinine)	AFC		Adjusted triclosan concentrations were inversely associated with AFC (*p:018*) in model. This association was modified by age and bodymass index, with the younger and leanerwomen showing larger decreases in AFC.
[39]	Urine and blood	PCOS: 30.4 ± 3.6	PCOS: 0.45 (0.25 – 1.54) ng/mL	LH, FSH, oestradiol and P4 for controls	OR: 1.99 (CI: 1.05 to 3.79), *p:0.0351*	Higher TCS level was associated with PCOS in infertile women.
		No-PCOS: 32.2 ± 4.2	No-PCOS: 0.37 (0.14 – 0.98) ng/mL			
[38]	Urine	29.8 ± 3.0	2.1 ng/mL (Median)		OR: 1.1 (CI: 0.95, 1.3), *p*>0.05	No association was observed between continuous triclosan concentrations and the risk of infertility. However, higher TCS concentrations were positively associated with risks of infertility when it was compared with low tertialof triclosan (*p=0.047*).

TCS: Triclosan; OR: odds ratios; PR: Prevalence Ratio: IC: confidence interval: CI; BPA: Bisphenol A; BP-3: Benzophenone-3; AFC: Antral follicle count; LH: Luteinizing hormone; FSH: follicle-stimulating hormone; AMH: Anti-Müllerian Hormone; PCOS: Polycystic ovary syndrome; LOD: Detection limit; E2: Estradiol; P4: progesterone; BMI: body mass index.
